# Cloning of a novel cyanobacterial serotonin N-acetyltransferase gene CySNAT3 and its integration with transcriptomic drug repurposing in pediatric obstructive sleep-disordered breathing

**DOI:** 10.3389/fphar.2026.1851793

**Published:** 2026-09-08

**Authors:** Wen Wen, Fei Xu, Yaoming Xu, Yifei Gao, Honglin Song, Yang Lv, Peng Yan

**Affiliations:** 1 School of Clinical Medicine, The First Affiliated Hospital of Chengdu Medical College, Chengdu, China; 2 Department of Neonatology, Shangrao Municipal Hospital, Shangrao, China; 3 Department of Pediatrics, Shangrao Municipal Hospital, Shangrao, China; 4 College of Chemistry and Materials Engineering, Quzhou University, Quzhou, China; 5 Department of Pediatrics, Rongchang District People’s Hospital, Chongqing, China; 6 Cardiovascular Department, The First Affiliated Hospital of Chengdu Medical College, Chengdu, China

**Keywords:** cyanobacteria, drug repurposing, melatonin, obstructive sleep-disordered breathing, serotonin N-acetyltransferase

## Abstract

**Background:**

Melatonin has potential therapeutic value in pediatric obstructive sleep-disordered breathing (oSDB). However, plant-based melatonin extraction faces limitations including long growth cycles, low yield, and complex purification procedures. Cyanobacteria, as photosynthetic microorganisms, offer distinct advantages such as rapid growth, low cultivation cost, and well-established genetic manipulation tools, making them ideal chassis cells for producing high-value natural products. Meanwhile, the molecular mechanisms linking tonsillar pathology to oSDB severity remain poorly understood, and translational therapeutic targets are urgently needed.

**Methods:**

A novel cyanobacterial serotonin N-acetyltransferase gene (CySNAT3) was cloned, expressed in *E. coli*, and purified by Ni-NTA affinity chromatography. Its enzymatic activity was assessed by HPLC-fluorescence. Separately, differential expression analysis, WGCNA, and drug repurposing were performed on tonsillar RNA-seq data (GSE274855), followed by molecular docking to validate drug-target interactions.

**Results:**

CySNAT3 catalyzed the conversion of serotonin to N-acetylserotonin and 5-MT to melatonin, providing an enzymatic tool for microbial melatonin production. Four high-confidence candidate drugs (sulfamethoxazole, cimetidine, diethylstilbestrol, clofibrate) were identified, with molecular docking confirming favorable binding affinities to their target proteins (CYP2C19, SLC47A1, WNT7A, SCD).

**Conclusion:**

Cimetidine and diethylstilbestrol have been experimentally linked to the melatonin pathway, suggesting that these four drugs may act through modulation of melatonin signaling. This study provides a new molecular foundation for pediatric oSDB treatment by integrating a novel cyanobacterial SNAT enzyme tool with transcriptome-derived drug candidates.

## 1 Introduction

Recurrent arousals caused by snoring during sleep disrupt normal sleep architecture and may lead to excessive daytime sleepiness, impaired attention, and an increased risk of cardiovascular and cerebrovascular complications (Zhao et al., 2025). These clinical manifestations are collectively referred to as sleep-disordered breathing (SDB). Based on pathophysiological mechanisms, SDB can be broadly classified into obstructive and central subtypes. Pediatric obstructive sleep-disordered breathing (oSDB) is a common condition in both pediatrics and otolaryngology–head and neck surgery, and has been associated with growth retardation, neurocognitive impairment, behavioral abnormalities, as well as cardiovascular, endocrine, and psychosomatic dysfunctions. If left untreated, oSDB may even increase the risk of sudden death in children (Ahmad et al., 2025). Therefore, early diagnosis and standardized treatment of oSDB are crucial for improving quality of life and long-term outcomes in affected children.

The prevalence of SDB in children is estimated to range from 10% to 17% (Rosen et al., 2003), among whom approximately 1%–3% progress to obstructive sleep apnea syndrome (OSAS) (Bonuck et al., 2015), making OSAS the second most common chronic health condition in childhood after asthma (Lumeng and Chervin, 2008; Oscullo et al., 2025). The risk of SDB increases during early childhood, coinciding with the peak age of physiological hypertrophy of the adenoids and tonsils (typically between 3 and 6 years of age). Adenotonsillar hypertrophy is a major contributing factor to upper airway obstruction in pediatric oSDB (Marcus et al., 2012; Kim et al., 2025).

Current management strategies for pediatric oSDB involve a multidisciplinary approach, including surgical intervention, medical therapy, and behavioral management (Arab et al., 2025; Ve et al., 2022). Adenotonsillectomy is considered the first-line surgical treatment for children with oSDB caused by adenotonsillar hypertrophy and can significantly alleviate clinical symptoms (Kang et al., 2021; De Sousa et al., 2025; Lildal et al., 2024). However, the rate of complete symptom resolution after surgery remains controversial, and the optimal timing of surgical intervention has not been definitively established. For children who are not suitable candidates for surgery or who continue to exhibit residual symptoms postoperatively, continuous positive airway pressure (CPAP) therapy is a well-established and effective non-surgical treatment option (Panetti et al., 2025; Saleh and Hasan, 2025; Bitners and Arens, 2020; Venekamp et al., 2015).

Despite the availability of multiple therapeutic approaches, the underlying pathogenesis of oSDB—particularly the role of focal infections and immune-related factors in disease initiation and progression—remains incompletely understood. Emerging evidence suggests that latent infections, such as Epstein–Barr virus (EBV), may contribute to oSDB by modulating local gene expression in the tonsils and altering the immune microenvironment of the upper airway (Sriram et al., 2025; Tonoyan et al., 2019). However, most previous transcriptomic studies have relied on peripheral blood samples rather than tonsillar tissue, which is more directly involved in the pathophysiology of upper airway obstruction. Notably, in pediatric patients, the tonsils represent a key target organ in the development of oSDB (Santos-Cortez et al., 2024). To date, systematic transcriptomic profiling of tonsillar tissue in both pediatric and adult oSDB remains relatively limited.

Melatonin were proved to be valuable for the treatment of pediatric obstructive sleep-disordered breathing (oSDB) (Wikner et al., 1997; Lasala and Lucero-Prisno, 2025; Bilyukov et al., 2015). Cyanobacteria, as photosynthetic microorganisms, present a compelling alternative to plant-based melatonin extraction. With fast proliferation (generation time 2–4 h), inexpensive culture conditions (requiring only light, CO_2_, and mineral salts), and robust genetic engineering systems, they serve as ideal chassis cells for producing high-value natural products (Kim et al., 2024; Agarwal et al., 2022).

SNAT is the key enzyme in the melatonin biosynthesis pathway, yet only one cyanobacterial SNAT gene has been cloned to date. To address this gap and extend our understanding of pediatric oSDB, we reanalyzed the publicly available dataset GSE274855 through integrated bioinformatic approaches—including differential expression analysis, functional annotation, and weighted gene co-expression network analysis (WGCNA)—to systematically investigate how EBV infection status and CPAP requirement shape the tonsillar transcriptome in pediatric oSDB. While the original publication primarily focused on identifying a limited set of differentially expressed genes and validating a single candidate (APOBR) across independent cohorts, our study adopts a systems-level, network-based framework to interrogate coordinated gene expression programs rather than isolated molecular changes. By integrating differential expression, WGCNA, hub gene prioritization, and drug repurposing analysis, we aimed to uncover higher-order regulatory modules and key molecular pathways associated with EBV infection and disease severity—an analytical depth not explored in the prior report.

Our objective was to identify core transcriptional signatures and pathway-level alterations linked to EBV status and clinical severity, and to explore potential therapeutic targets through computational drug repurposing. These findings are expected to provide novel insights into the molecular mechanisms by which EBV contributes to the pathogenesis of pediatric oSDB and may ultimately inform more personalized treatment strategies.

## 2 Materials and methods

### 2.1 Identification of CySNAT3 gene

The protein sequence of OsSNAT3 was used as a query to perform a BLASTP search against cyanobacterial protein sequences in the NCBI database. The hit with the highest score was designated as the candidate gene.

### 2.2 Production of CySNAT recombinant proteins in *Escherichia coli*

The coding DNA sequence (CDS) of CySNAT3 was amplified by PCR using the genomic DNA of cyanobacterium PCC8603 as a template. The primers were listed in Supplementary Table S1. The amplified CDS was then inserted into the *Saccharomyces cerevisiae* SUMO (psumo) vector linearized with SacI or into the pET-32a vector linearized with EcoRI. The resulting recombinant vectors were transformed into *E. coli* BL21 (DE3) (TransGen Biotech, Beijing, China). To produce the recombinant CySNAT3 protein, the transformed *E. coli* were first cultured on Luria-Bertani (LB) agar plates (10 g/L tryptone, 5 g/L yeast extract, 10 g/L NaCl, 15 g/L agar) supplemented with 100 mg/L ampicillin. A single colony was then picked and inoculated into 5 mL of LB broth (10 g/L tryptone, 5 g/L yeast extract, 10 g/L NaCl) containing 100 mg/L ampicillin, followed by overnight incubation at 37 °C. Subsequently, 3 mL of this overnight culture was inoculated into 300 mL of fresh LB broth with the same concentrations of antibiotics (100 mg/L ampicillin) and grown until the optical density at 600 nm (OD600) reached 0.6–0.8. Protein expression was induced by adding 100 µM isopropyl-β-D-thiogalactopyranoside (IPTG; Sigma, St. Louis, MO, United States of America), and the culture was further incubated at 16 °C with shaking at 160 rpm for 22 h. The cells were then disrupted using a high-pressure homogenizer, and the subsequent purification steps were performed using Ni-NTA resin according to the manufacturer’s instructions (Vazyme, Nanjing, China; NEB, Beijing, China). The purified protein was concentrated using an Amicon Ultra-4 centrifugal filter (Merck Millipore, Carrigtwohill, Ireland) and finally dissolved in 20 mM Tris-HCl (pH 8.0).

### 2.3 Measurement of SNAT enzyme activity

For the enzymatic activity assay, 10 µg of purified recombinant SNAT protein was used. The reaction was performed in a total volume of 100 µL containing 0.5 mM serotonin or 5-MT as the substrate and 0.5 mM acetyl-CoA in 20 mM Tris-HCl buffer (pH 7.4). The mixture was incubated at 45 °C for 1 h, and the reaction was terminated by adding 200 µL methanol (MeOH). The reaction mixture was then centrifuged at 10,000 rpm for 2 min, and the supernatant was filtered through a 0.22 µm PTFE membrane. A 5 µL aliquot was injected into a high-performance liquid chromatography (HPLC) system equipped with a fluorescence detector (LC-20, Shimadzu) to quantify N-acetylserotonin (NAS) or melatonin. Chromatographic separation was achieved on an Ultimate XB-C18 column (4.6 * 150 mm; Welch, Shanghai, China) at a flow rate of 1 mL/min using an isocratic elution with 30% MeOH in 0.1% formic acid over 20 min. NAS and melatonin were detected at excitation and emission wavelengths of 280 nm and 348 nm, respectively. All measurements were performed in triplicate. Non-enzymatic reactions were used as the controls (containing only the substrate and 0.5 mM acetyl-CoA, without the recombinant CySNAT3 protein).

### 2.4 Data acquisition and preprocessing

Transcriptomic data used in this study were obtained from the Gene Expression Omnibus (GEO) database under accession number GSE274855. This dataset contains mRNA expression profiles derived from tonsillar tissue samples of pediatric patients diagnosed with obstructive sleep-disordered breathing (oSDB). Samples were categorized based on EBV detection status in the tonsils (positive or negative) and clinical requirement for continuous positive airway pressure (CPAP) therapy, resulting in four phenotypic groups: EBV + CPAP+, EBV + CPAP−, EBV−CPAP+, and EBV−CPAP−. Raw expression matrices were downloaded from the GEO database and normalized using the DESeq2 package to ensure comparability across samples.

### 2.5 Differential expression analysis

To investigate the independent effects of EBV infection and CPAP requirement on the tonsillar transcriptome, two separate differential expression analyses were performed, using EBV status (EBV + vs. EBV−) and CPAP requirement (CPAP + vs. CPAP−) as grouping variables, respectively. Differentially expressed genes (DEGs) were identified using the DESeq2 package. The threshold for significance was set at an absolute log2 fold change (|log_2_FC|) ≥ 1 and a p-value <0.05. Visualization of DEGs was performed using the ggplot2 and pheatmap packages.

### 2.6 Functional enrichment and pathway analysis

Gene Ontology (GO) and Kyoto Encyclopedia of Genes and Genomes (KEGG) pathway enrichment analyses were conducted for the identified DEGs using the clusterProfiler package. The human genome annotation database (OrgDb: org. Hs.e.g.,.db) was used as the background reference. Enrichment significance was assessed using a hypergeometric test, followed by false discovery rate (FDR) correction for multiple comparisons. Terms with an FDR <0.05 were considered statistically significant.

### 2.7 Weighted gene co-expression network analysis

The union of DEGs identified from the EBV- and CPAP-based comparisons was used as input for weighted gene co-expression network analysis (WGCNA). An unsigned gene co-expression network was constructed using the WGCNA package in R. Soft-thresholding power was selected based on scale-free topology criteria. Genes with highly correlated expression patterns were clustered into modules using hierarchical clustering and dynamic tree cutting, with each module assigned a unique color identifier. Module eigengenes (MEs) were calculated to represent the principal expression profile of each module. Pearson correlation analysis was performed to assess the associations between MEs and external clinical traits (EBV status and CPAP requirement), enabling the identification of modules most strongly related to specific phenotypes.

### 2.8 Identification of hub genes

Weighted gene co-expression network analysis (WGCNA) was performed to identify gene modules associated with clinical phenotypes. Hub genes were selected using a dual criterion: absolute Module Membership (KME) > 0.6 and absolute Gene Significance (GS) > 0.3.

### 2.9 Drug repurposing analysis

Integrated drug–gene interaction data were retrieved from the Drug–Gene Interaction Database (DGIdb). Hub genes associated with CPAP requirement and EBV infection were used as target gene sets and matched against DGIdb to identify known drug–gene interaction pairs.

### 2.10 Drug prioritization scoring system

To systematically evaluate the repurposing potential of candidate drugs, we developed a multidimensional prioritization algorithm integrating four core components:

Interaction Quality Score: Calculated as log1p (n_interactions) × mean_score, this metric rewards both the breadth and strength of drug-gene interactions, with logarithmic transformation mitigating the diminishing returns of high interaction counts.

Target Importance Score: Derived from the mean Module Membership (KME) and Gene Significance (GS) values of target hub genes, this component reflects the topological centrality and phenotypic relevance of drug targets within the disease-associated co-expression networks.

Module Coverage Bonus: A weighted coefficient assigned based on module specificity: 1.5 for dual-module targeting (simultaneous engagement of both CPAP and EBV modules), 1.0 for single-module targeting, and 0.8 for non-module-associated interactions, prioritizing drugs capable of intervening in independent pathological pathways.

Approval Status Bonus: A 1.2-fold weight applied to clinically approved drugs (vs. 1.0 for non-approved compounds), incorporating translational feasibility by favoring agents with established safety profiles and known pharmacokinetic properties.

The comprehensive repurposing score was calculated as the product of these four components:

Repurposing Score_raw = Interaction Quality * Target Importance * Module Bonus * Approval Bonus.

Raw scores were subsequently normalized to a 0–10 scale to facilitate cross-drug comparison:

RepurposingScore = 10 * (RepurposingScore_raw/max (RepurposingScore_raw))

This multiplicative framework ensures synergistic integration of multidimensional criteria, prioritizing candidates with robust interaction evidence, high target centrality, multi-module coverage, and clinical approval status for subsequent experimental validation.

### 2.11 Confidence tier classification

High Tier: Repurposing score ≥0.5 + approved status + at least 2 target hub genes; Medium Tier: Repurposing score ≥0.3 or approved status; Low Tier: All remaining drugs.

### 2.12 Data visualization

All visualizations were generated using ggplot2 (version 3.4.0), pheatmap (version 1.0.12), and patchwork (version 1.1.2) packages. Heatmap clustering was performed using Ward’s minimum variance method.

### 2.13 Molecular docking

Molecular docking was employed to predict the binding modes between small molecules (ligands) and target proteins (receptors, such as enzymes or transcription factors). In this study, a semi-flexible docking strategy was adopted to simulate the formation of stable ligand–receptor complexes, which is critical for elucidating molecular mechanisms and screening potential lead compounds. Docking calculations were performed using AutoDock Vina version 1.2.7 (Trott and Olson, 2010; Eberhardt et al., 2021), where diethylstilbestrol (PubChem CID: 448537) was docked with WNT7A (AlphaFold ID: AF-O00755-F1-v6) and CYP2C19 (PDB ID: 4GQS), sulfamethoxazole (PubChem CID: 5329) was docked with SLC47A1 (AlphaFold ID: AF-Q96FL8-F1-v6) and CYP2C19 (PDB ID: 4GQS), cimetidine (PubChem CID: 2756) was docked with SLC47A1 (AlphaFold ID: AF-Q96FL8-F1-v6) and CYP2C19 (PDB ID: 4GQS), and clofibrate (PubChem CID: 2796) was docked with SCD (AlphaFold ID: AF-O00767-F1-v6) and CYP2C19 (PDB ID: 4GQS). Protein structures were preprocessed using PyMOL version 3.0.3, including removal of crystallographic water molecules and non-relevant ligands, followed by the addition of polar hydrogen atoms. The ligand structure was prepared and energy-minimized using ChemDraw 20.0. Subsequently, AutoDock Tools version 1.5.7 was used to convert both protein and ligand structures into the PDBQT format. Potential binding pockets of the receptor were predicted using Cyscore (Cao and Li, 2014), with the default setting generating five candidate binding pockets. Molecular docking was then performed independently for each pocket, producing nine binding conformations per pocket. The binding affinities of all docking poses were evaluated, and the pocket exhibiting the lowest binding energy among the five candidates was selected as the most probable ligand-binding site. Finally, docking results were visualized using PyMOL version 3.0.3 to illustrate the binding conformations and intermolecular interactions between the ligand and the target protein. Two-dimensional interaction diagrams were generated using Discovery Studio.

## 3 Results

### 3.1 Identification of CySNAT3 gene

**FIGURE 1:**
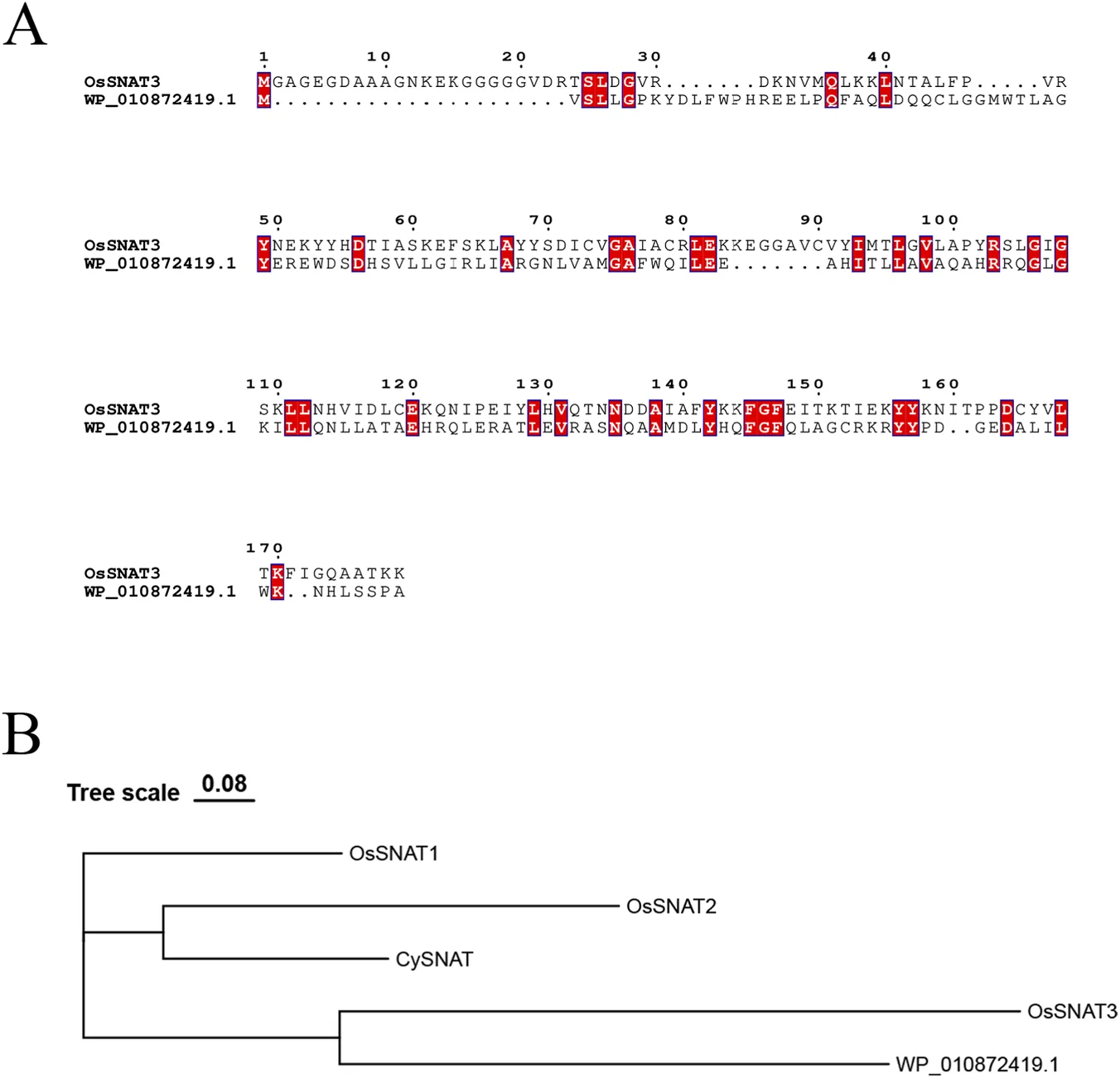
Comparison of CySNAT3 with OsSNAT3 and other SNAT homologs **(A)** Sequence alignment of CySNAT3 and OsSNAT3 **(B)** Phylogenetic tree of CySNAT3, OsSNAT3 and other cloned SNAT proteins.

By searching against cyanobacterial protein sequences using OsSNAT3 as a query, WP_010872419.1, which showed the highest sequence similarity to OsSNAT3, was identified as the candidate gene for cyanobacterial SNAT3 (Figure 1A). Phylogenetic tree analysis revealed that this gene clustered together with OsSNAT3 in the same clade, indicating a close evolutionary relationship (Figure 1B).

### 3.2 Functional validation of CySNAT3

**FIGURE 2:**
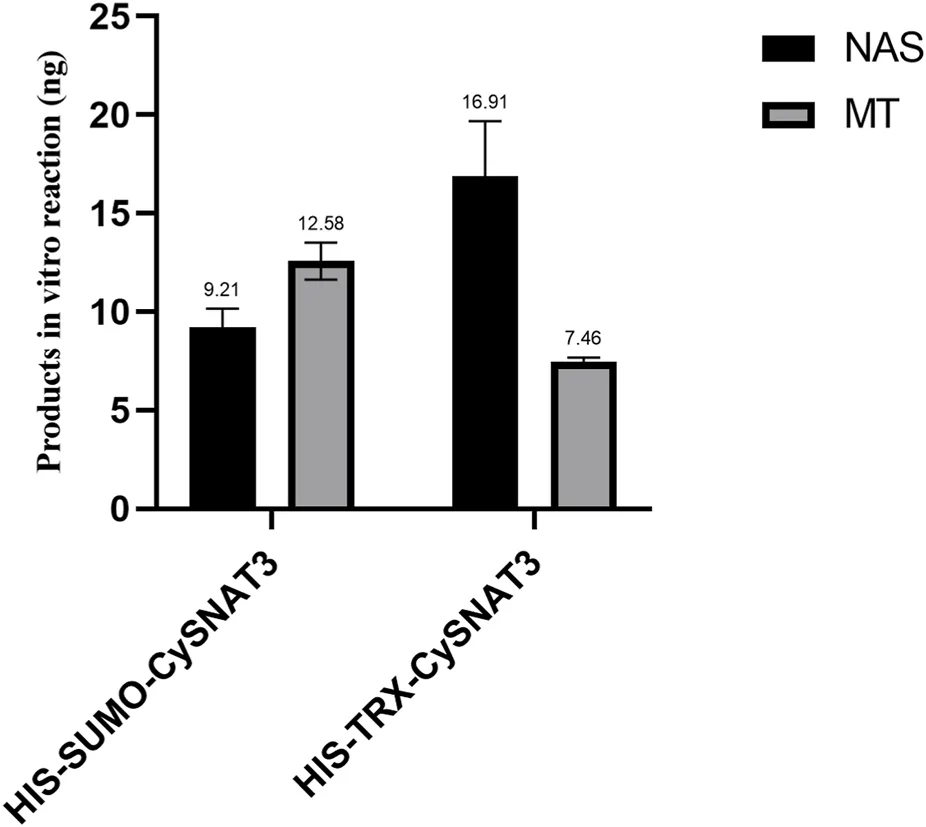
Enzymatic activity of the recombinant CySNAT3, with the substrate of serotonin and 5-MT. The reaction mixture contained 10 μg purified recombinant protein, 0.5 mM substrate (serotonin or 5-MT), and 0.5 mM acetyl-CoA, incubated at 45 °C for 1 h; all measurements were performed in three independent replicates (n = 3), and data are presented as mean ± SD. Non-enzymatic reactions (without recombinant protein) were used as negative controls.

By employing fusion tags including HIS-SUMO and His-Trx-tag, we obtained soluble CySNAT3 fusion proteins. *In vitro* enzymatic activity assays confirmed that both recombinant proteins were capable of catalyzing the conversion of serotonin to N-acetylserotonin (NAS) and also converting 5-methoxytryptamine (5-MT) to melatonin (Figure 2). These results demonstrate that the CySNAT3 gene was successfully cloned and the encoded protein exhibits SNAT activity.

### 3.3 Dataset characteristics and sample grouping

The present analysis was based on the GEO dataset GSE274855, which includes tonsillar tissue samples from 16 pediatric patients with obstructive sleep-disordered breathing (oSDB). Based on EBV detection in the tonsils, samples were divided into an EBV-positive group (EBV+, n = 7) and an EBV-negative group (EBV−, n = 9). According to clinical evaluation of disease severity and treatment requirements, samples were further categorized into a CPAP-recommended group (CPAP+, n = 3) and a non-CPAP group (CPAP−, n = 13).

### 3.4 EBV infection–associated differentially expressed genes and pathway enrichment

**FIGURE 3:**
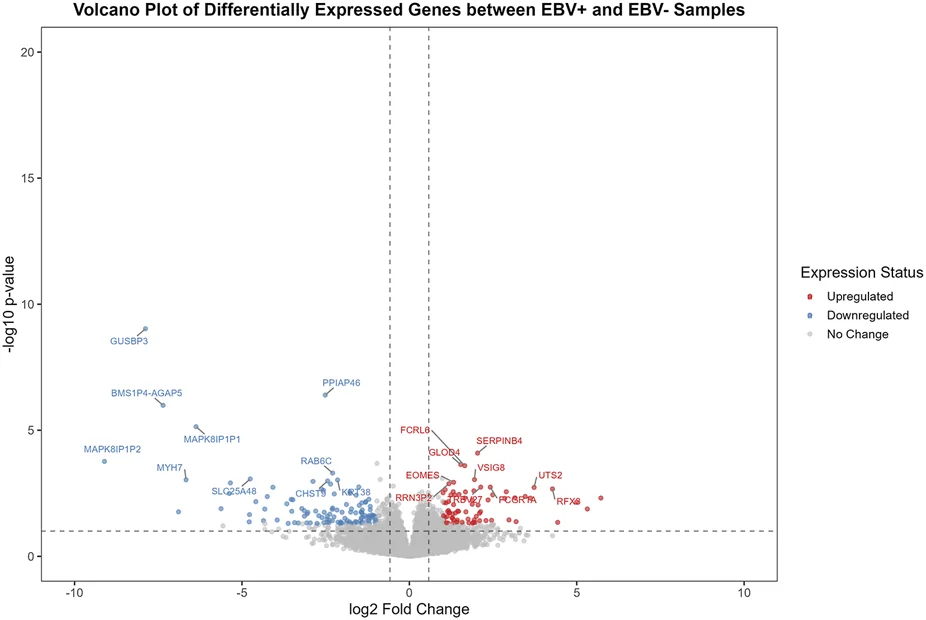
Volcano plot of differentially expressed genes between EBV-positive and EBV-negative groups.

To systematically identify molecular alterations associated with EBV infection, differential expression analysis was performed between the EBV+ and EBV− groups. Using a threshold of |log_2_FC| ≥ 1 and p-value <0.05, a total of 190 differentially expressed genes (DEGs) were identified, including 75 upregulated and 115 downregulated genes (Figure 3).

**FIGURE 4:**
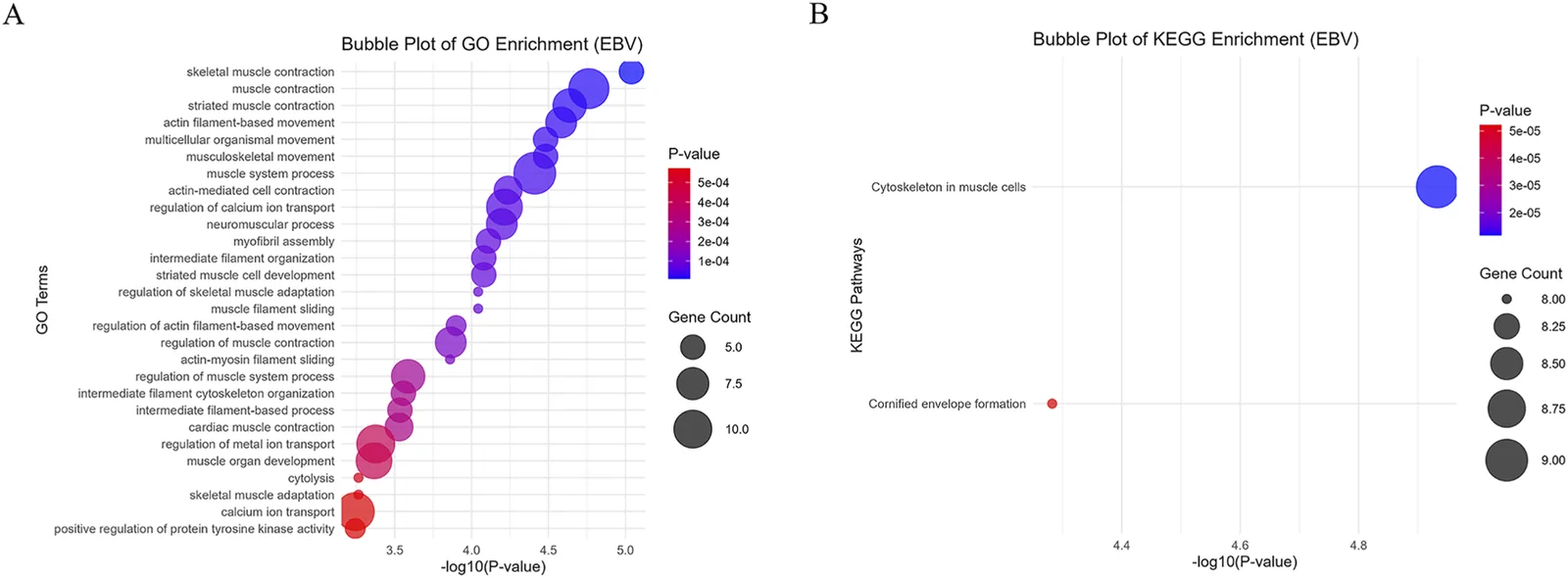
GO and KEGG enrichment analysis of differentially expressed genes between EBV-positive and EBV-negative groups **(A)** GO enrichment analysis **(B)** KEGG enrichment analysis.

To further elucidate the biological processes potentially involved in EBV-associated genes, Gene Ontology (GO) enrichment analysis was performed. EBV-related differentially expressed genes (DEGs) were predominantly enriched in biological processes associated with myosin binding, cytoskeletal organization, and ion transport (Figure 4A). The most significantly enriched terms included skeletal muscle contraction, muscle contraction, actin filament-based movement, and regulation of calcium ion transport.

These findings indicate that the transcriptional impact of EBV infection in tonsillar tissue extends beyond classical antiviral immune responses, involving structural and biomechanical processes. Notably, several genes traditionally associated with muscle contractility, such as MYH7, TNNC1, and STC1, exhibited altered expression levels, suggesting a potential role for EBV in modulating tissue mechanics.

KEGG pathway enrichment further supported these observations, revealing significant enrichment in cytoskeleton in muscle cells and cornified envelope formation pathways (Figure 4B). Collectively, these results suggest that EBV infection may contribute to the development and progression of pediatric oSDB by altering the biomechanical properties of tonsillar tissue, rather than solely through immune-mediated inflammation.

### 3.5 Differential expression and pathway enrichment associated with CPAP treatment requirement

**FIGURE 5:**
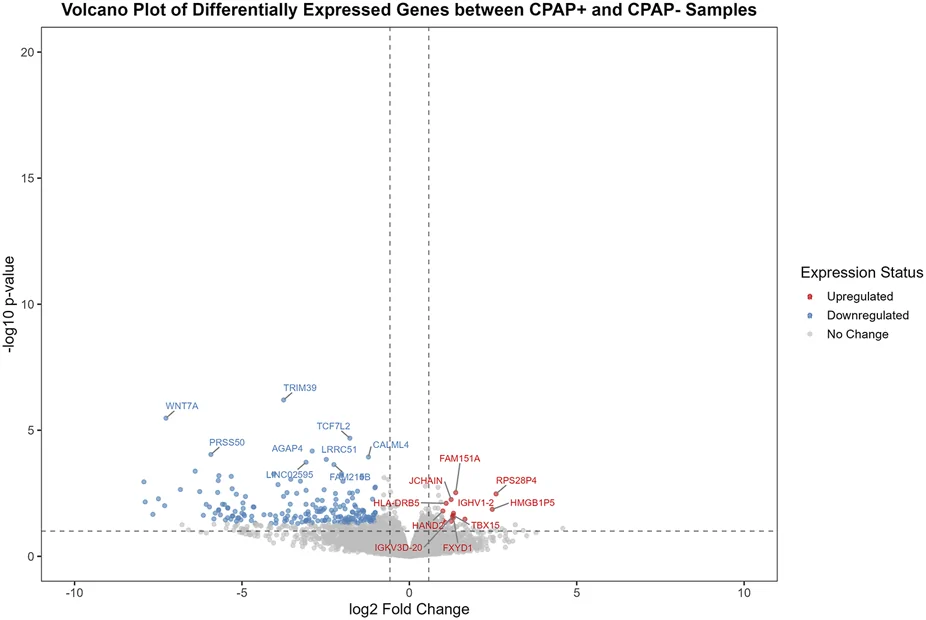
Volcano plot of differentially expressed genes between CPAP+ and CPAP- groups.

Using the same statistical thresholds, a total of 205 DEGs were identified between the CPAP+ and CPAP− groups, including 14 upregulated and 191 downregulated genes (Figure 5).

**FIGURE 6:**
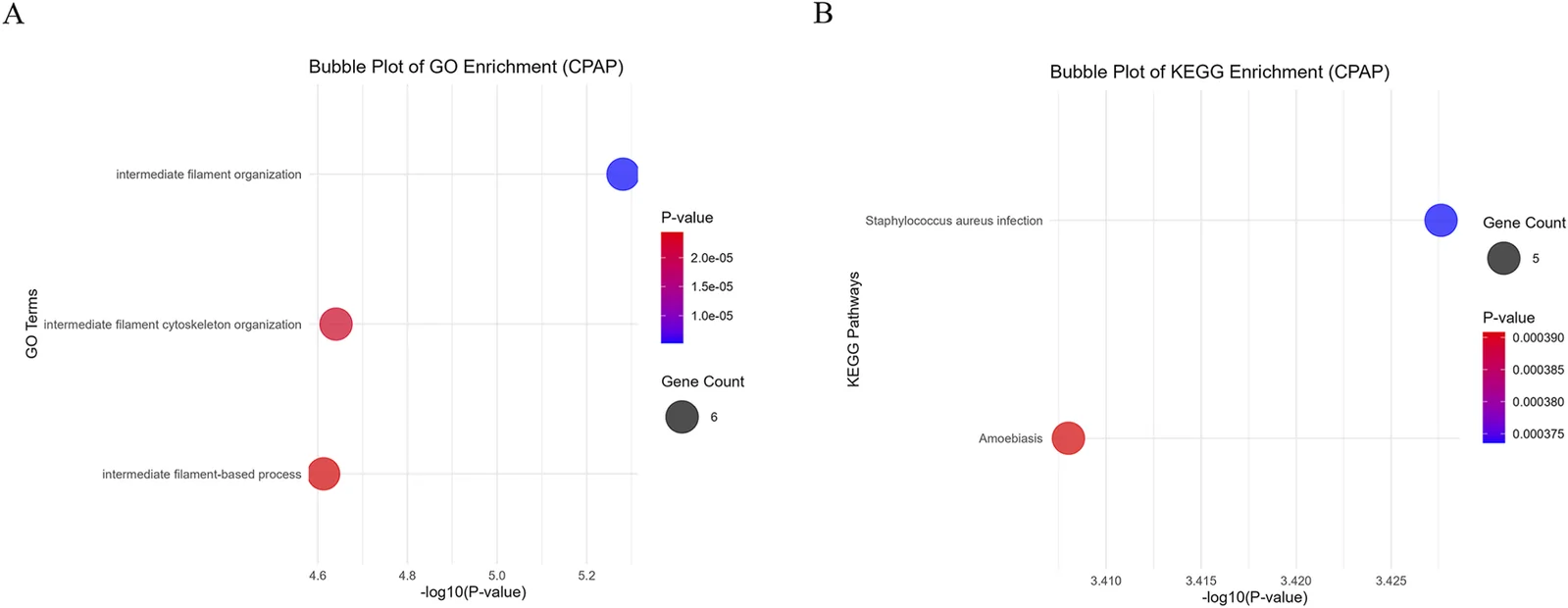
GO and KEGG enrichment analysis of differentially expressed genes between CPAP+ and CPAP- groups **(A)** GO enrichment analysis **(B)** KEGG enrichment analysis.

GO analysis revealed that CPAP-recommended DEGs were strongly enriched in pathways related to intermediate filament composition and organization, including intermediate filament organization, intermediate filament cytoskeleton organization, and intermediate filament-based processes (Figure 6A). These findings suggest pronounced cytoskeletal remodeling and altered mechanical properties in tonsillar tissues from children with more severe oSDB. Specifically, reprogramming of epithelial (keratin) and mesenchymal (vimentin) structural proteins may lead to increased tissue stiffness and reduced elasticity, thereby exacerbating upper airway resistance.

KEGG pathway analysis further identified enrichment in *Staphylococcus aureus* infection and amoebiasis pathways (Figure 6B), implying that severe oSDB may be associated with both structural tissue remodeling and a chronic low-grade inflammatory state.

### 3.6 Identification of co-expression modules associated with clinical traits by WGCNA

**FIGURE 7:**
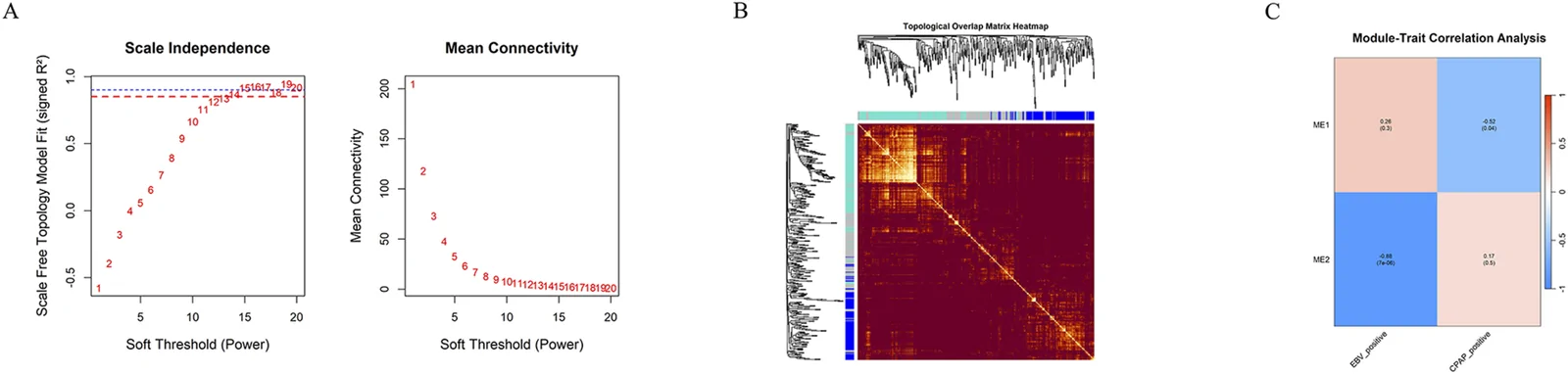
Soft-threshold power selection and module-trait relationship analysis **(A)** Soft-threshold power selection. The red dashed line indicates R^2^ = 0.85, and the blue dashed line indicates R^2^ = 0.9 **(B)** Heatmap of module-trait relationships **(C)** Heatmap of module-trait relationships.

To focus on biologically relevant genes potentially linking viral infection and disease severity, the union of DEGs from both comparisons was used as the input gene set for downstream network analysis. Importantly, this intersected gene set served as a foundation for constructing co-expression networks and did not imply that all subsequent hub genes would be differentially expressed in both phenotypic comparisons. To move beyond single-gene analyses, weighted gene co-expression network analysis (WGCNA) was applied to explore gene expression patterns at the network level. A soft-thresholding power of 14 was selected, achieving a scale-free topology fit index (R^2^) of 0.85 (Figure 7A). Two distinct gene co-expression modules were identified: a turquoise module (ME1, 153 genes) and a blue module (ME2, 113 genes) (Figure 7B).

Module–trait relationship analysis demonstrated that the blue module (ME2) was strongly negatively correlated with EBV positivity (r = −0.88, p = 7 × 10^−6^), whereas the turquoise module (ME1) showed a significant negative correlation with CPAP treatment requirement (r = −0.52, p = 0.04) (Figure 7C).

### 3.7 Identification of hub genes and high-confidence candidate drugs

**FIGURE 8:**
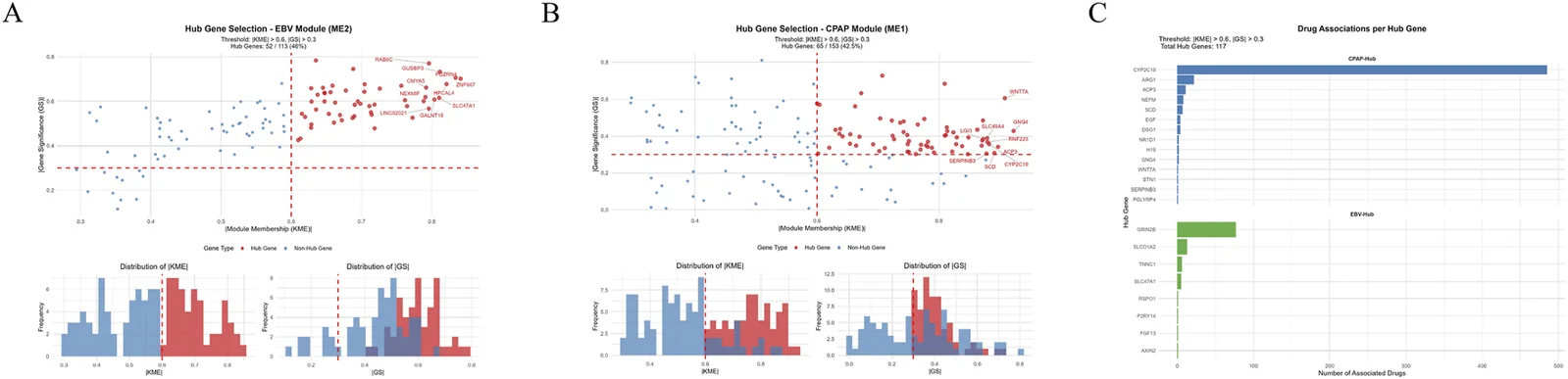
Classification and distribution of hub genes and drugs in pediatric oSDB-associated modules **(A)** The distribution of 52 hub genes within the EBV-associated module (113 genes) **(B)** The distribution of 65 hub genes within the CPAP-recommended module (153 genes) **(C)** Bar plot of drug association counts for core hub genes.

A dual criterion of |KME| > 0.6 and |GS| > 0.3 to screen hub genes was employed. From the EBV infection-associated blue module (113 genes), 52 hub genes were identified (46.0% of the module), with a mean KME of 0.726 and a mean |GS| of 0.604, including ZNF667, PDZRN4, among others (Figure 8A). From the CPAP-recommended turquoise module (153 genes), 65 hub genes were screened (42.5% of the module, with no overlap with the EBV-related module), exhibiting a mean KME of 0.761 and a mean |GS| of 0.498, including GNG4, WNT7A, among others (Figure 8B; Supplementary Tables S2-S5). The absence of overlapping hub genes between the two modules suggests partially distinct transcriptional patterns underlying CPAP requirement and EBV infection.

Drug–gene interaction analysis based on the Drug–Gene Interaction Database (DGIdb) revealed that the 117 hub genes (65 CPAP-related +52 EB V-related) corresponded to 627 potential therapeutic drugs, of which 271 (43.2%) were clinically approved medications. Specifically, CPAP hub genes were associated with 551 interaction records and 537 unique drugs (averaging 8.48 drugs per gene), with CYP2C19 exhibiting the highest number of interacting drugs (485 drugs), corresponding to 249 approved drugs (covering 13.8% of CPAP hub genes). EBV hub genes were associated with 117 interaction records and 101 unique drugs (averaging 2.25 drugs per gene), with GRIN2B showing the highest number of interacting drugs (88 drugs), corresponding to 30 approved drugs, covering 7.7% of EBV hub genes (Figure 8C). Notably, only eight approved drugs simultaneously targeted both modules (3.0% of approved drugs) (Supplementary Table S6).

### 3.8 Identification of high-confidence drug candidates through multi-criteria prioritization

**FIGURE 9:**
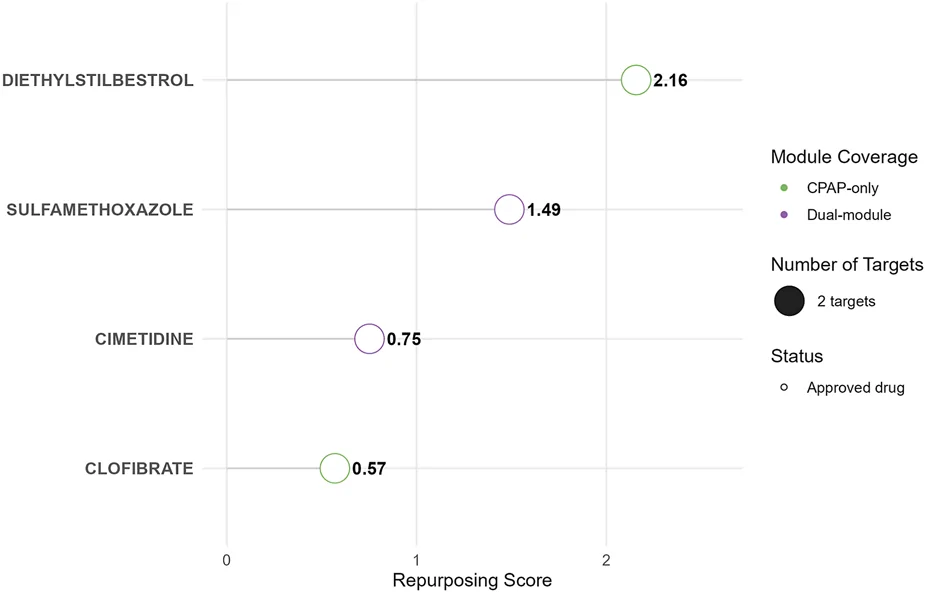
Characteristics of high tier candidate drugs.

**FIGURE 10:**
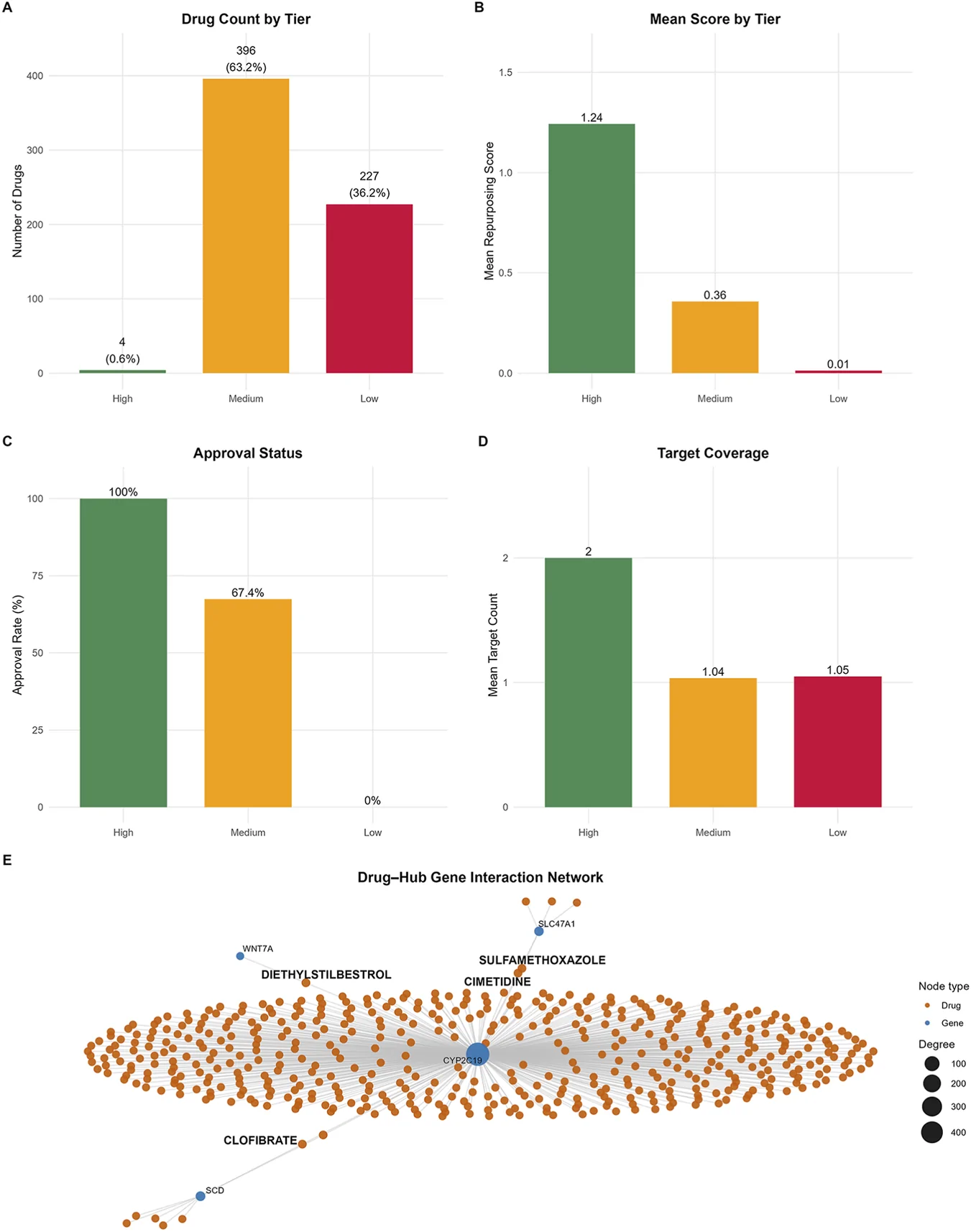
Comparative analysis of confidence tiers and drug-hub gene interaction network **(A)** Bar plot showing the number of drugs in each confidence tier. The high tier contains 4 drugs (0.6%), the medium tier contains 396 drugs (63.2%), and the low tier contains 227 drugs (36.2%) **(B)** Bar plot of the mean repurposing score by tier. High tier drugs exhibit the highest mean score (1.24), followed by Medium (0.36) and Low (0.01) tiers **(C)** Bar plot of approval status by tier. High tier drugs are 100% approved, compared to 67.4% in the medium tier and 0% in the low tier **(D)** Bar plot of mean target count per drug by tier. High tier drugs target an average of 2.0 hub genes, whereas Medium and low tier drugs target approximately 1.04 genes **(E)** Drug-hub gene interaction network. Blue nodes represent genes, orange nodes represent drugs. Node size reflects connectivity and edges indicate drug-gene interactions.

Stringent confidence tier classification criteria was employed: high tier drugs were required to meet a score ≥0.5, target at least 2 high-confidence targets, and be approved drugs. Among the 627 candidate drugs, only 4 (0.6%) met the high tier criteria (Figure 9; Figure 10A). These four drugs exhibited excellent comprehensive characteristics: all were clinically approved, targeted an average of 2 hub genes, and achieved a mean interaction score as high as 0.803 (Supplementary Table S7). Notably, the CYP2C19 gene emerged as a common target across all high tier drugs, highlighting its central role in the pathophysiology of oSDB. As a key member of the cytochrome P450 family, CYP2C19 encodes a drug-metabolizing enzyme and exhibited a mean interaction score of 1.594 across the four high tier drugs, indicating a strong association with drug action.

Score distribution analysis revealed that high tier drugs had a mean repurposing score of 1.24, higher than the mean score of 0.36 for medium tier and 0.01 for low tier drugs (Figure 10B). High tier drugs outperformed other drugs across multiple key metrics: 100% approval rate compared to 67.4% of medium tier drugs (Figure 10C) and greater number of targets (2.0 vs. 1.04) (Figure 10D). Drug interaction analysis showed sulfamethoxazole and cimetidine, as dual-module targeting drugs, simultaneously acted on the CYP2C19 gene in the CPAP module and the SLC47A1 gene in the EBV module, providing clues for understanding the cross-mechanisms between the two independent pathological modules (Figure 10E).

Interaction score analysis revealed that diethylstilbestrol exhibited an exceptionally high interaction score of 3.182 with the WNT7A gene, far exceeding other drug-gene pairs, suggesting that this estrogen-like drug may play an important role in oSDB treatment through modulation of the Wnt signaling pathway. WNT7A, as the second-ranked hub gene in the CPAP module (KME = 0.908, GS = −0.605), is involved in tissue development and repair processes and is highly correlated with the pathological mechanism of tonsillar hypertrophy (Supplementary Table S7).

Module specificity analysis demonstrated that diethylstilbestrol and clofibrate specifically targeted the CPAP module, while sulfamethoxazole and cimetidine achieved dual-module coverage. This finding aligns with the independence of pathological mechanisms suggested by previous studies and provides a basis for combination therapy strategies. Particularly, sulfamethoxazole, as a widely used antibiotic, has demonstrated immunomodulatory properties and may exert multifaceted therapeutic effects through simultaneous targeting of metabolic pathways (CYP2C19) and solute transporters (SLC47A1).

### 3.9 Molecular docking predicts drug-target binding affinities

To further explore the drug-target interactions predicted by drug repurposing analysis, molecular docking to assess the binding affinities between the four high tier candidate drugs (cimetidine, clofibrate, diethylstilbestrol, sulfamethoxazole) and their respective target gene-encoded proteins (CYP2C19, SLC47A1, WNT7A, SCD) were employed.

**FIGURE 11:**
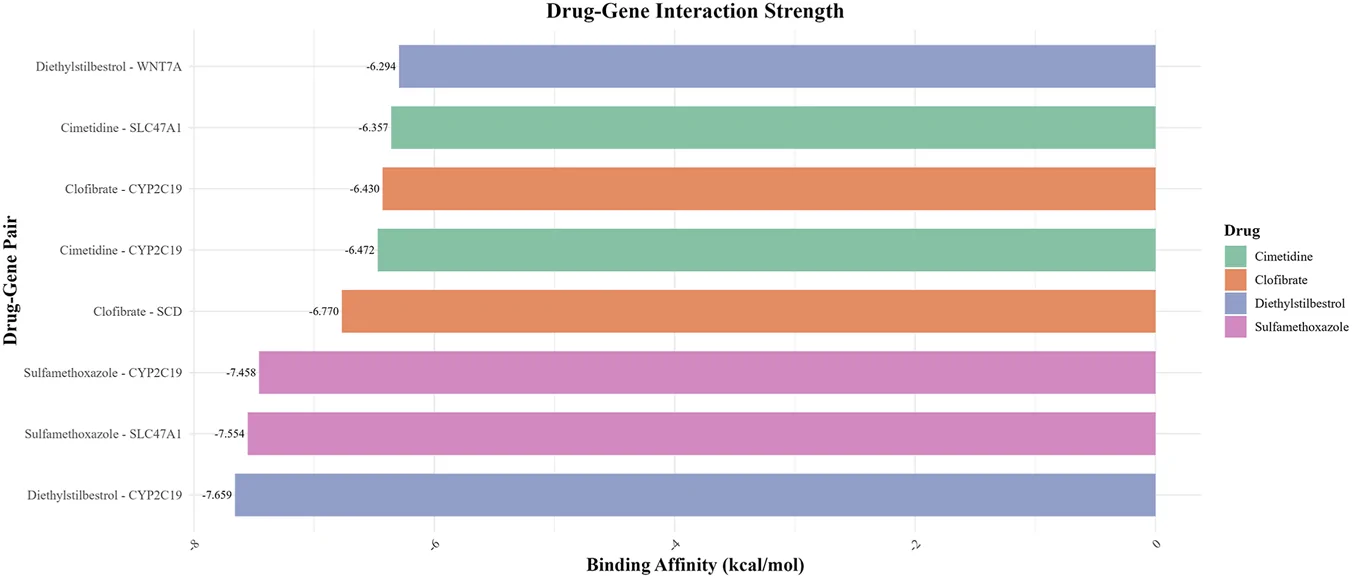
Bar plot showing molecular docking binding affinities of high-tier drugs against target proteins.

Molecular docking results predicted that all drug-target pairs may exhibit favorable binding affinities, with binding energies ranging from −7.659 to −6.294 kcal/mol (Figure 11). Notably, diethylstilbestrol showed the strongest binding affinity with CYP2C19, achieving a binding energy of −7.659 kcal/mol, indicating a highly stable interaction between this estrogen-like drug and the metabolic enzyme CYP2C19. Sulfamethoxazole also exhibited excellent binding characteristics, with binding energies of −7.458 kcal/mol and −7.554 kcal/mol for CYP2C19 and SLC47A1, respectively, providing structural basis for its dual-module targeting capability (Figure 11).

Remarkably, all drugs demonstrated binding energies below −6.43 kcal/mol with CYP2C19 (−7.659 to −6.43 kcal/mol), further confirming the central role of CYP2C19 as a core drug target. Cimetidine exhibited a binding energy of −6.357 kcal/mol with SLC47A1, and clofibrate showed a binding energy of −6.77 kcal/mol with SCD, both below the −6.0 kcal/mol threshold, suggesting potentially relevant interactions that warrant experimental verification.

**FIGURE 12:**
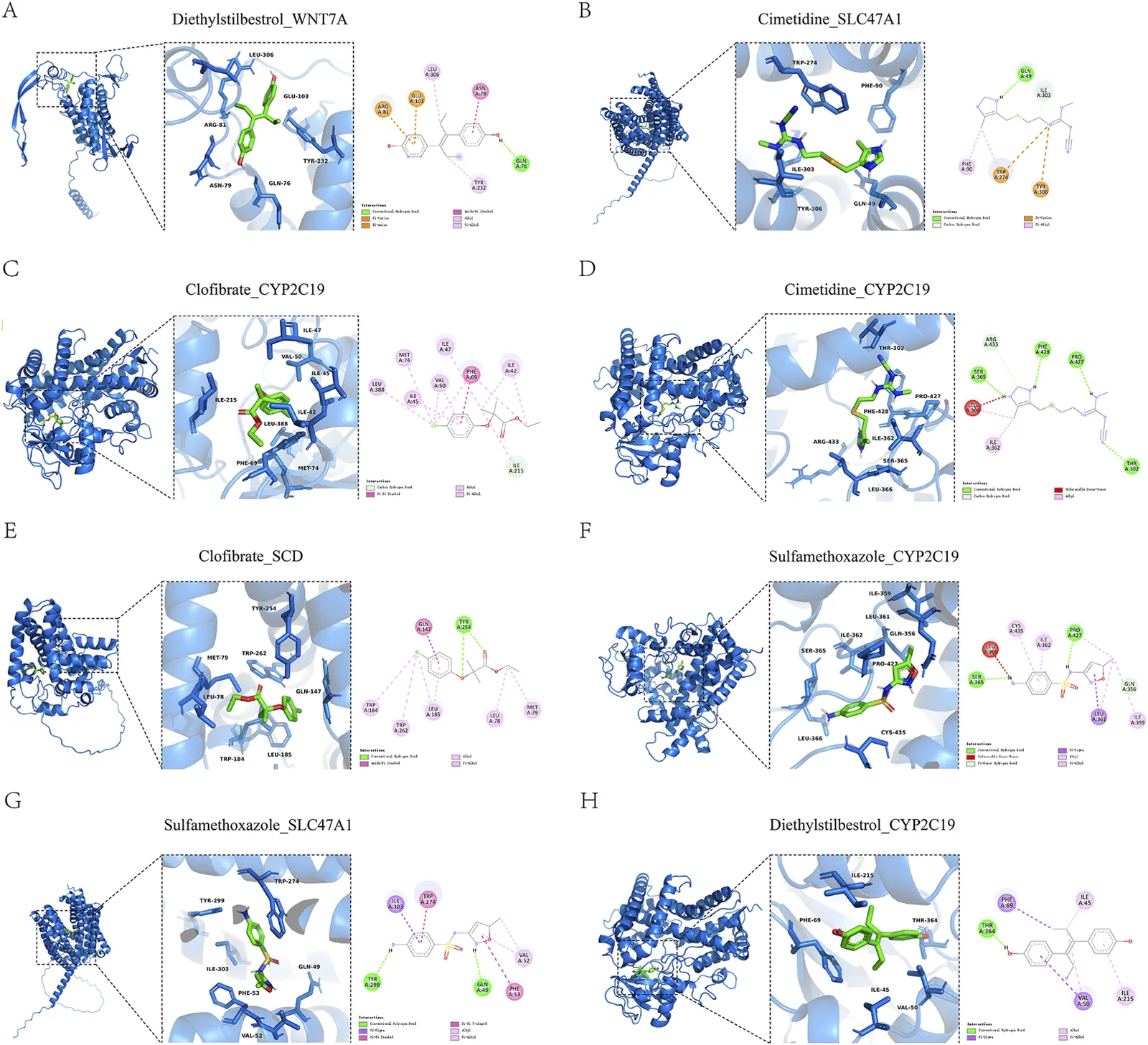
Molecular docking analysis of high tier candidate drugs with target proteins. The figure displays docking results for eight representative drug-protein pairs **(A)** Diethylstilbestrol-WNT7A **(B)** Cimetidine-SLC47A1 **(C)** Clofibrate-CYP2C19 **(D)** Cimetidine-CYP2C19 **(E)** Clofibrate-SCD **(F)** Sulfamethoxazole-CYP2C19 **(G)** Sulfamethoxazole-SLC47A1, and **(H)** Diethylstilbestrol-CYP2C19. For each panel, the left image shows the overall docking conformation with the protein represented as a cartoon model and the ligand as sticks; the middle image provides a close-up view of the binding site highlighting key amino acid residues; and the right image depicts a 2D schematic diagram of ligand-protein interactions, including hydrogen bonds, hydrophobic contacts, and π-π stacking interactions.

Detailed analysis of the docking conformations revealed the molecular basis of these interactions (Figures 12A–H). In the diethylstilbestrol-CYP2C19 complex (Figure 12H), the drug molecule deeply embeds into the hydrophobic active pocket of CYP2C19, forming multiple hydrophobic interactions and hydrogen bonds with surrounding amino acid residues, explaining its excellent binding energy. The binding mode of sulfamethoxazole with CYP2C19 (Figure 12F) shows that its benzene ring structure forms π-π stacking interactions with aromatic amino acids in the active site, while the sulfonamide group establishes a hydrogen bond network with key residues. Docking results of sulfamethoxazole with SLC47A1 (Figure 12G) reveal that the drug can form a stable complex with the substrate-binding domain of the transporter. The binding of cimetidine to SLC47A1 (Figure 12B) is primarily achieved through hydrogen bonds and hydrophobic interactions, while the binding of clofibrate to SCD (Figure 12E) depends on its hydrophobic tail embedding into the hydrocarbon chain binding channel of the enzyme.

These molecular docking results predict favorable interactions between the four high-tier candidate drugs and their target proteins, providing structural hypotheses for their potential application in pediatric oSDB treatment and supporting subsequent experimental validation.

## 4 Discussion

In this study, we successfully cloned a novel serotonin N-acetyltransferase gene (CySNAT3) from cyanobacteria and confirmed its function through heterologous expression in *E. coli* and *in vitro* enzymatic assays. To the best of our knowledge, only one cyanobacterial SNAT gene has been reported in the literature to date. CySNAT3 represents the second functionally validated SNAT from cyanobacteria, enriching the microbial resource pool of melatonin biosynthesis enzymes.

Compared with previously reported plant SNATs, cyanobacterial SNAT offers distinct technical advantages. Plant SNATs are typically localized in chloroplasts, contain signal peptides, and exhibit codon bias, often leading to the formation of inactive inclusion bodies when expressed in *E. coli*. In contrast, as a prokaryote, cyanobacteria possess SNAT genes with simple structures and no introns, allowing efficient expression as soluble and active proteins in prokaryotic systems. In this study, we obtained soluble recombinant proteins using two different fusion tags (HIS-SUMO and His-Trx-tag), both of which exhibited catalytic activity, further demonstrating the favorable heterologous expression characteristics of cyanobacterial SNAT. One limitation of this study is that we only expressed the recombinant cyanobacterial SNAT in *E. coli* and validated its *in vitro* activity; we have not yet over-expressed this gene in a cyanobacterial host to examine its impact on endogenous melatonin production.

Systems-level, network-based transcriptomic framework to interrogate tonsillar RNA-seq data from a clinically well-characterized pediatric oSDB cohort was applied in this study. Rather than replicating previously reported differential expression analyses, our approach was designed to extend gene-level observations by identifying coordinated transcriptional programs and central regulatory genes associated with EBV infection and disease severity. This integrative strategy provides mechanistic insight into how local tonsillar biology may contribute to heterogeneous clinical phenotypes in pediatric oSDB.

A principal finding of this analysis is that EBV infection and disease severity are governed by largely distinct co-expression networks, despite arising within the same tissue context. The EBV-associated module was significantly enriched for transcriptional regulators, Wnt/β-catenin signaling components, and calcium signaling genes. This pattern suggests that EBV infection may exert its effects through coordinated reprogramming of intracellular signaling and tissue organization, rather than through diffuse, nonspecific inflammatory responses. Such network-level modulation offers a plausible molecular explanation for clinical observations linking EBV positivity to altered sleep physiology in the absence of overt inflammatory manifestations (Feuth, 2024; Lupo et al., 2023; Besedovsky et al., 2019).

Notably, enrichment of cytoskeletal and muscle contraction-related pathways in EBV-associated tonsils introduces a previously underappreciated dimension of EBV pathobiology in oSDB. While previous studies have primarily focused on the immunomodulatory role of EBV, associating it with local inflammatory cell infiltration and cytokine release, the present study reveals for the first time that EBV infection is associated with alterations in cytoskeletal dynamics and contractile gene expression (Draborg et al., 2016; Ouaguia et al., 2014; Ryan et al., 2012). Alterations in actin dynamics, calcium handling, and contractile gene expression may reflect changes in tissue composition—for example, differences in stromal versus epithelial cell proportions—rather than direct measurement of tissue biomechanics. These transcriptional signatures warrant further investigation using histological or imaging approaches. This conceptual framework reframes EBV from a purely immunological trigger to a modulator of tissue mechanics and airway function, expanding our understanding of viral contributions to sleep-disordered breathing.

In contrast, the turquoise module, which was negatively correlated with CPAP recommendation (i.e., enriched in non-CPAP/less severe cases), was characterized by a transcriptional network involved in cytoskeletal organization and lipid metabolism. This suggests that downregulation of these structural and metabolic genes may be associated with more severe disease, potentially reflecting loss of tissue integrity or metabolic decompensation in progressive airway obstruction. This finding contrasts with prior studies, which have largely attributed oSDB severity to anatomical adenotonsillar hypertrophy or systemic inflammatory responses, whereas our study uncovers an independent contribution of local tissue mechanical properties and metabolic reprogramming (Ko et al., 2003; Hu et al., 2025; Marchica et al., 2019; Apnea, 2024). The involvement of lipid metabolism regulators, particularly SCD, in the module associated with less severe disease is noteworthy. While obesity and metabolic dysfunction are established risk factors for severe oSDB, our data suggest that transcriptional downregulation of SCD and related lipid metabolism genes may accompany disease progression, warranting further mechanistic investigation.

A key methodological insight from this study is that biologically meaningful hub genes are not necessarily those exhibiting the largest or most consistent fold changes across phenotypic contrasts. In this analysis, hub genes were defined by intramodular connectivity and network centrality within trait-associated modules derived from the union of EBV- and CPAP-related differentially expressed genes. Consequently, some hub genes were not differentially expressed in both phenotypic comparisons. Rather than representing a limitation, this observation underscores the strength of network-based approaches in identifying regulatory genes that coordinate disease-relevant transcriptional programs (Langfelder and Horvath, 2008; Pei et al., 2017; Zhang and Wong, 2022).

Leveraging these network-derived hub genes, we further explored the translational relevance of our findings through drug repositioning analysis. The identification of four clinically approved compounds—sulfamethoxazole, cimetidine, diethylstilbestrol, and clofibrate—as high-confidence candidates highlights the therapeutic potential of targeting metabolic and structural pathways in pediatric oSDB. These drugs are already widely used in clinical practice: sulfamethoxazole as a first-line antibacterial agent for respiratory tract infections (Madhi et al., 2023; Eliopoulos and Huovinen, 2001), cimetidine as a histamine H2 receptor antagonist for gastric acid suppression (Okabe et al., 1977; Nakano et al., 2011), diethylstilbestrol as a synthetic estrogen previously widely used for hormone replacement therapy (Giusti et al., 1995; Watkins, 2007; Herbst and Anderson, 2015), and clofibrate as a lipid-lowering agent for regulating lipid metabolism (Kesäniemi and Grundy, 1984; Oyagbemi et al., 2022; Sun et al., 2025). Molecular docking validated their binding affinities to respective target proteins (CYP2C19, SLC47A1, WNT7A, and SCD), with sulfamethoxazole and cimetidine demonstrating dual-module targeting capability. The established clinical safety profiles and well-characterized pharmacokinetic properties of these drugs support their translational feasibility for potential application in oSDB treatment. While these results remain exploratory and require experimental validation, they demonstrate how network-guided prioritization can accelerate hypothesis generation and therapeutic discovery.

Cimetidine has been reported to increase serum melatonin concentration by approximately 1.7-fold through inhibition of CYP450 1A2 and 2D6-mediated hepatic metabolism (Andersen et al., 2016), which may theoretically enhance melatonin’s anti-inflammatory, antioxidant, and sleep-regulating effects by increasing its bioavailability–although this hypothesis requires validation in oSDB-specific models. Diethylstilbestrol, a synthetic estrogen, has been shown to interact with melatonin in animal studies, with melatonin counteracting its effects in the rat anterior pituitary (Zhao et al., 2010); however, analogous mechanisms in oSDB tonsillar tissue remain unproven. For sulfamethoxazole and clofibrate, no direct evidence links them to the melatonin pathway in the current literature–their potential association is purely speculative based on target functions. Taken together, these connections are derived from literature inference rather than direct experimental evidence from the present study, and warrant confirmation or refutation in future mechanistic investigations.

In summary, the existing evidence for cimetidine and DES supports the potential value of the melatonin pathway in pediatric oSDB treatment and provides preliminary experimental rationale for the hypothesis of synergistic effects between melatonin and the four drugs proposed in this study.

## 5 Conclusion

This study cloned and validated a novel cyanobacterial SNAT gene (CySNAT3), providing an enzymatic tool for microbial melatonin production. WGCNA of pediatric oSDB tonsillar transcriptomes revealed that EBV infection and disease severity are driven by two independent co-expression modules, enriched in signaling/tissue remodeling and cytoskeleton/lipid metabolism pathways, respectively. Drug repurposing based on network hub genes identified four high-confidence candidates (sulfamethoxazole, cimetidine, diethylstilbestrol, clofibrate), of which cimetidine and diethylstilbestrol have been experimentally linked to the melatonin pathway. In summary, this study provides new molecular tools and candidate drugs for the treatment of pediatric oSDB, laying a foundation for future research.

## Data Availability

The original contributions presented in the study are included in the article/Supplementary Material, further inquiries can be directed to the corresponding authors.

## Supplementary material

SUPPLEMENTARY TABLE S1 List of primers used for the amplification and vector insertion of the CySNAT3 CDS.

SUPPLEMENTARY TABLE S2 Hub Genes in the EBV-Associated Module (ME2).

SUPPLEMENTARY TABLE S3 Hub Genes in the CPAP-recommended Module (ME1).

SUPPLEMENTARY TABLE S4 Hub genes in the EBV-associated module identified by |KME|>0.6 and |GS|>0.3 criteria.

SUPPLEMENTARY TABLE S5 Hub genes in the CPAP-recommended module identified by |KME|>0.6 and |GS|>0.3 criteria.

SUPPLEMENTARY TABLE S6 Drug repurposing priority scores and confidence tier classification for all candidate drugs.

SUPPLEMENTARY TABLE S7 Prioritized drug candidates for pediatric oSDB repurposing.

SUPPLEMENTARY TABLE S8 Grid-box coordinates and dimensions.
